# Conserved phosphorylation sites in the activation loop of the *Arabidopsis* phytosulfokine receptor PSKR1 differentially affect kinase and receptor activity

**DOI:** 10.1042/BJ20150147

**Published:** 2015-11-27

**Authors:** Jens Hartmann, Dennis Linke, Christine Bönniger, Andreas Tholey, Margret Sauter

**Affiliations:** * Entwicklungsbiologie und Physiologie der Pflanzen, Christian-Albrechts-Universität Kiel, Am Botanischen Garten 5, 24118 Kiel, Germany; † Systematische Proteomics und Bioanalytik, Christian-Albrechts-Universität Kiel, Niemannsweg 11, 24105 Kiel, Germany

**Keywords:** activation loop, *Arabidopsis thaliana*, leucine-rich receptor receptor-like kinase, peptide signalling, phytosulfokine, PSKR1

## Abstract

PSK (phytosulfokine) is a plant peptide hormone perceived by a leucine-rich repeat receptor kinase. Phosphosite mapping of epitope-tagged PSKR1 (phytosulfokine receptor 1) from *Arabidopsis thaliana* plants identified Ser^696^ and Ser^698^ in the JM (juxtamembrane) region and probably Ser^886^ and/or Ser^893^ in the AL (activation loop) as *in planta* phosphorylation sites. *In vitro*-expressed kinase was autophosphorylated at Ser^717^ in the JM, and at Ser^733^, Thr^752^, Ser^783^, Ser^864^, Ser^911^, Ser^958^ and Thr^998^ in the kinase domain. The LC–ESI–MS/MS spectra provided support that up to three sites (Thr^890^, Ser^893^ and Thr^894^) in the AL were likely to be phosphorylated *in vitro*. These sites are evolutionarily highly conserved in PSK receptors, indicative of a conserved function. Site-directed mutagenesis of the four conserved residues in the activation segment, Thr^890^, Ser^893^, Thr^894^ and Thr^899^, differentially altered kinase activity *in vitro* and growth-promoting activity *in planta*. The T899A and the quadruple-mutated TSTT-A (T890A/S893A/T894A/T899A) mutants were both kinase-inactive, but PSKR1(T899A) retained growth-promoting activity. The T890A and S893A/T894A substitutions diminished kinase activity and growth promotion. We hypothesize that phosphorylation within the AL activates kinase activity and receptor function in a gradual and distinctive manner that may be a means to modulate the PSK response.

## Abstract (precis)

Phytosulfokine is perceived by a leucine-rich repeat receptor-like kinase with auto- and *trans*-phosphorylation activity. Phosphosite mapping indicated that multisite serine/threonine autophosphorylation probably occurs within the activation loop of the kinase. Phosphoablative mutations differentially impair kinase activity *in vitro* and receptor function *in planta*.

## INTRODUCTION

The pentapeptide PSK (phytosulfokine) with the amino acid backbone YIYTQ has been described as a peptide hormone that promotes plant growth through elevated cell expansion. Furthermore, its involvement in the response to pathogens has been reported [1–7]. Peptide activity depends on post-translational sulfation of the two tyrosine residues which is catalysed by the enzyme tyrosylprotein sulfotransferase in the *trans*-Golgi network [8].

**Figure 1:**
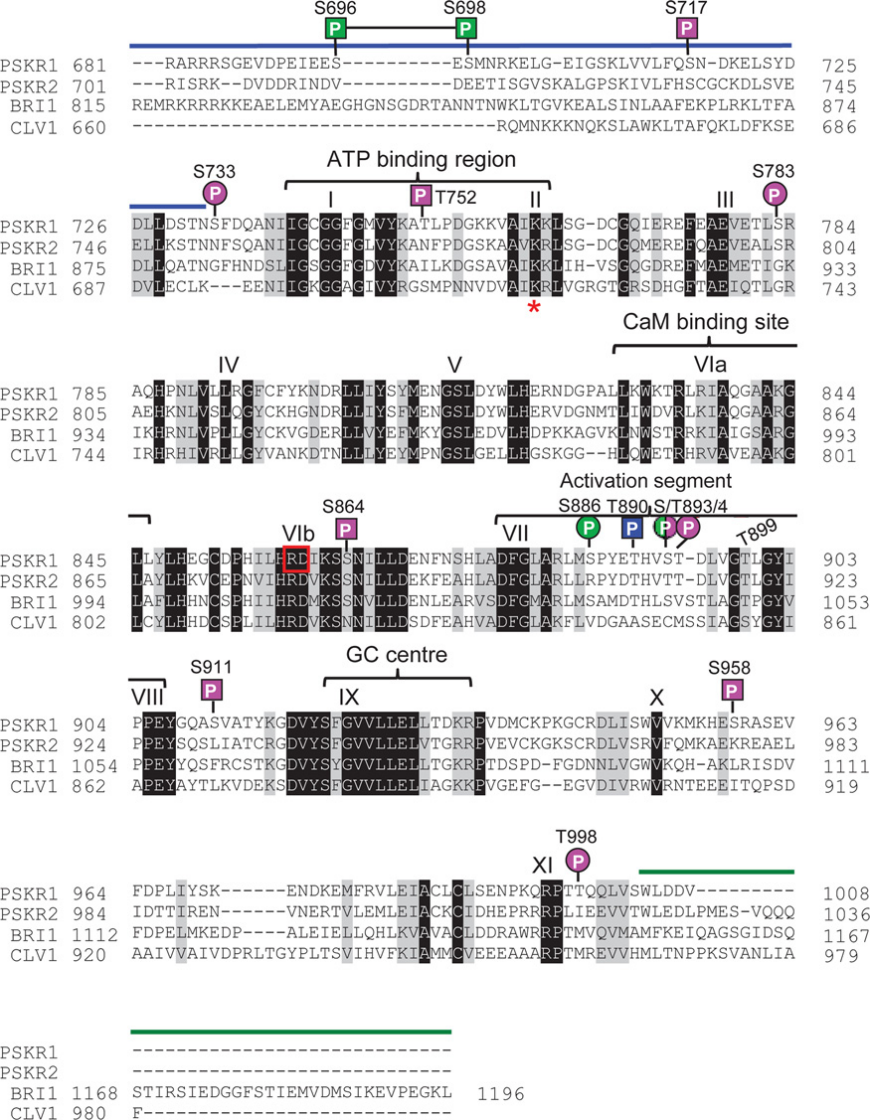
The cytoplasmic domain of the LRR RLK PSKR1 of *Arabidopsis* indicating conserved subdomains, guanylate cyclase centre, calmodulin-binding site and phosphorylation sites A ClustalW multiple alignment of the cytoplasmic domains of PSKR1, PSKR2 and the related RD kinases BRI1 and CLV1 from *Arabidopsis* was carried out. The PSKRs belong to the class of RD kinases with a designated AL. The JM domain of PSKR1 from amino acids 681–732 is indicated by a blue line. The KD of PSKR1 with its 12 subdomains starts at Ser^733^ and ends at Ser^1003^ (for a definition of the PSKR1 kinase subdomains, see [14,15]). An invariant lysine residue (Lys^762^ in PSKR1) in the ATP-binding site located in subdomain II is indicated by a red asterisk. The invariant Arg^859^ and Asp^860^ residues in subdomain VIb are boxed in red. Indicated also are the calmodulin (CaM)-binding site, the AL and the designated guanylate cyclase (GC) centre. The C-terminal region is indicated by a green line. The short C-terminus of PSKR1 begins with amino acid 1004. Unambiguous phosphorylation sites (P) of PSKR1 are marked with squares; unconfirmed phosphorylation sites are designated by a circle. Pink indicates *in vitro* sites and green indicates *in planta* sites. Shown in blue is a site that is also phosphorylated in *E. coli*.

Perception and transduction of the PSK signal requires a membrane-localized receptor protein. PSK is the natural ligand for the PSK receptor kinases PSKR1 and PSKR2 in *Arabidopsis thaliana*. PSKR1 and PSKR2 belong to a large monophyletic gene family of *Arabidopsis* LRR RLKs (leucine-rich repeat receptor-like kinases) [9–11]. LRR RLKs are characterized by a defined organization of functional domains: LRRs with an extracellular ligand-binding domain, a single transmembrane domain and a cytoplasmic protein portion consisting of a catalytic KD (kinase domain) flanked by two regulatory sequences, the JM (juxtamembrane) region and a CT (C-terminal) domain. PSKR1 shares this organization of functional domains. PSKR1 possesses a predicted extracellular domain with 21 LRRs. Between the 17th and 18th LRR of PSKR1 a 36-amino-acid island domain is located that was shown to bind PSK [9,12,13]. A single helical 21-amino-acid transmembrane domain spans the plasma membrane followed by a JM region, a serine/threonine KD with its 12 conserved subdomains and a short CT domain [9,14–16] (Figure 1).

PSK perception at the cell surface probably triggers a cellular auto- and *trans*-phosphorylation cascade. It has been shown that PSKR1 kinase activity is essential for growth promotion via PSKR1 signalling, although the targets that are recognized and phosphorylated by the activated PSKR1 *in planta* are not known [16]. Ligand-induced receptor dimerization followed by autophosphorylation of the cytoplasmic domain is considered a conserved mechanism of receptor kinase action in plants and was shown for the brassinosteroid receptor BRI1 (BRASSINOSTEROID-INSENSITIVE 1) that is closely related to PSKRs [17–19]. PSKR1, on the other hand, oligomerizes even in the absence of ligand and autophosphorylates *in vitro* [16,20]. Phosphorylation of the cytoplasmic domain of receptor kinases can occur on multiple sites in the JM region, KD and CT domain [21]. The structure of serine/threonine protein KDs is highly conserved, whereas the JM and CT regions are less conserved. The protein structure can be separated into two domains: a small N-terminal ATP-binding domain consisting of five β-sheets and one α-helix (αC) and a larger C-terminal substrate-binding domain that is predominantly helical [21,22].

A conserved activation segment with the central AL (activation loop) is located in the C-terminal lobe within the kinase subdomains VII and VIII as shown for the PSKRs BRI1 and CLV1 (CLAVATA1) (Figure 1). PSKRs belong to the RD-type kinases. An invariant aspartate residue in subdomain VIb (Asp^860^ in PSKR1) is required for catalytic activity and can be found in all kinases. In RD kinases, this aspartate residue is preceded by a positively charged arginine residue (Arg^859^ in PSKR1) (Figure 1). Ligand-induced kinase activation of some, but not all, RD kinases necessitates phosphorylation of one to three residues in the AL [22]. In these cases, the conserved arginine residue probably interacts with phosphorylated negatively charged amino acids of the AL, resulting in charge neutralization and substrate access to the catalytic aspartate residue [23].

Compared with the KDs, the JM region and the CT domain show little sequence conservation among receptor kinases. Nonetheless, these domains were shown to regulate activity of BRI1 [24,25]. The phosphorylation state of the non-catalytic JM and CT regions of BRI1 is important not only for modulating kinase activity, but also for determining substrate specificity. It is conceivable that the JM region and CT domain of PSKR1 have similar functions in defining PSKR1 substrates and/or receptor activity. Neither the positions of phosphorylation nor their role in PSK signalling have been described to date.

To elucidate PSKR1 function, it is essential to understand early molecular events of PSKR1 signalling such as receptor modification. In the present study, we used LC–ESI–MS/MS analysis of the recombinant PSKR1 KD to show that the cytoplasmic protein portion of the receptor is autophosphorylated on specific serine and threonine residues *in vitro*. Furthermore, phosphosite mapping was performed to identify phosphorylation sites of PSKR1 *in planta*. We studied further the functional role of identified phosphorylation sites in the PSKR1 AL with respect to *in vitro* kinase activity. Additionally, the contribution of phosphorylation of specific PSKR1 AL residues to receptor function *in planta* was assessed by overexpressing site-mutated PSKR1 variants in the receptor-null background. Our data support the view that site-directed phosphoablative mutagenesis of specific PSKR1 AL residues impairs PSKR1 kinase activity *in vitro* and that phosphorylation of these serine and threonine residues is likely to be required for growth signalling through PSKR1 *in planta*.

## EXPERIMENTAL

### Plant material, growth conditions and transformation

All experiments were carried out with *Arabidopsis thaliana* (L.) Heynh. ecotype Columbia (Col-0). For plant growth, a 2:3 sand/humus mixture was frozen at −80°C for 2 days to avoid insect contamination. For experiments under sterile conditions, seeds were surface-sterilized for 20 min in 1 ml of 2% (w/v) sodium hypochlorite (NaClO) followed by five washing steps with autoclaved water and laid out on half-concentrated modified Murashige and Skoog medium [26] and 1.5% (w/v) sucrose, solidified with 0.38% Gelrite™ (Duchefa Biochemie) and supplemented with 1 μM PSK as indicated (NeoMPS). Seeds were stratified at 4°C in the dark for 2 days and then transferred to 22°C with a 16 h light (70 μM photons·m^−2^·s^−1^)/8 h dark cycle.

The *pskr1-2 pskr2-1* double knockout line was described previously [3,4]. Experiments were performed with homozygous plants as tested by spraying with 200 μM glufosinate ammonium (Basta, AgrEvo). *Arabidopsis* plants were transformed with *Agrobacterium tumefaciens* (GV3101) using the floral dip method [27]. Transgenic plants were selected by spraying with 200 μM Basta. Expression of transgenes was verified by RT (reverse transcription)–PCR.

### Growth measurements and statistical analysis

The software ImageJ (NIH) was used to determine root lengths from photographs. The open source program Rosette Tracker [28] was used to determine projected rosette areas from photographs. The program Minitab (http://www.minitab.com) was used for statistical analysis. Unless stated otherwise, statistical significance of means was tested by an ANOVA (Tukey's test) or a two-sample Student's *t* test. Constant variance and normal distribution of data were verified before statistical analysis. If one of these conditions was not achieved, the *P* value was set to <0.001. *P* values for the Pearson product moment correlation are indicated in Figure legends. Kruskal–Wallis all-pairwise comparisons of kinase mutants were performed for autophosporylation and for *trans*-phosphorylation activities.

### Site-directed mutagenesis of the PSKR1 full-length and cytoplasmic domain coding sequence and cloning of constructs

Site-directed mutagenesis of the cytoplasmic domain of PSKR1 was performed by overlap-extension PCR [29]. Five constructs with the following substitutions were generated: T890A, T890D, T899A, S893A/T894A and TSTT-A (T890A/S893A/T894A/T899A). The encoded proteins encompassed the intracellular protein part of PSKR1 from amino acid 686 to 1008. To replace each specific serine or threonine with alanine or aspartate, primers 5′-GTCCTTACGAGGCTCATG-TAAGTAC-3′ and 5′-GTACTTACATGAGCCTCGTAAGGAC-3′ for T890A, primers 5′-GTCCTTACGAGGATCATGTAA-GTAC-3′ and 5′-GTACTTACATGATCCTCGTAAGGAC-3′ for T890D, primers 5′-CTGATTTGGTTGGAGCTTTAGGT-TAC-3′ and 5′-GTAACCTAAAGCTCCAACCAAATCAG-3′ for T899A, primers 5′-ACGCATGTAGCTGCTGATTTG-GTTG-3′ and 5′-CAACCAAATCAGCAGCTACATGCGT-3′ for S893A/T893A and primers 5′-CGAGGCTCATGTA-GCTGCTGATTTGGTTGGAGCTTTAG-3′ and 5′-CTAAAG-CTCCAACCAAATCAGCAGCTACATGAGCCTCG-3′ for TSTT-A were used for *in vitro* mutagenesis. PCR products were cloned into pETDuet-1 (Merck) resulting in His_6_ N-terminal fusions with the five mutant PSKR1 kinases.

Point-mutated T890A, T890D, T899A, S893A/T894A and TSTT-A full-length *PSKR1* sequences were generated by overlap-extension PCR using the primers described above. The PCR fragments were cloned into the vector pB7WG2.0 downstream of the CaMV (cauliflower mosaic virus) 35S promoter using the Gateway cloning system (Life Technologies). All constructs were sequenced to verify the specific mutations and to exclude unwanted mutations. The C-terminal in-frame fusion of GFP with PSKR1 and cloning of the PSKR1–GFP construct into the vector pB7WG2.0 downstream of the CaMV 35S promoter were performed as described previously [16].

### Recombinant protein expression, purification and *in vitro* kinase assay

Constructs of the five mutant PSKR1 kinases T890A, T890D, T899A, S893A/T894A and TSTT-A were transformed into *Escherichia coli* BL21(DE3) cells (Life Technologies). Protein expression was induced by adding 1 mM IPTG (Sigma–Aldrich) followed by incubation of bacterial cultures for 16 h at 20°C. Soluble proteins were purified at native conditions on a TALON column (Clontech) as described in the manufacturer's instructions and stored at 4°C.

*In vitro* kinase activity was assayed in 50 mM Hepes (pH 7.4), 10 mM MnCl_2_, 1 mM DTT, 0.2 mM unlabelled ATP, 20 μCi of [γ-^32^P]ATP, 0.25 μg of affinity-purified His_6_–PSKR1-KD mutant and 0.5 μg of MBP (myelin basic protein) in a final volume of 8 μl at 25°C for 1 h followed by the addition of 4× SDS loading buffer to stop reactions. Proteins were separated by SDS/PAGE (15% gel), stained with Coomassie Blue, dried and exposed to an X-ray film. The protein bands were excised, mixed with Ultima Gold (PerkinElmer) and radioactivity was determined by liquid-scintillation counting in a Tri-Carb 2910 TR instrument (PerkinElmer). The background signal of the gel was subtracted.

### Immunoprecipitation and immunoblot analysis

GFP-tagged full-length PSKR1 protein was extracted from 14-day-old *35S:PSKR1-GFP* and wild-type seedlings. The plant material was ground in liquid nitrogen and the tissue was extracted in 1 ml of extraction buffer per g of plant material. The extraction buffer contained 50 mM Tris/HCl (pH 7.5), 150 mM NaCl and 1% (v/v) Igepal CA 630 (Sigma–Aldrich). After incubation of protein samples on ice for 1 h, suspensions were centrifuged at 300 ***g*** for 3 min at 4°C. The protein concentration was determined with Roti®-Quant (Carl Roth). Then 10 ml (1 mg/ml) of total plant protein in extraction buffer was incubated with 100 μl of 50% (v/v) Protein A–Sepharose CL-4B beads (GE Healthcare) in 50 mM Tris/HCl (pH 7.5) and 150 mM NaCl. The samples were centrifuged at 300 ***g*** for 3 min at 4°C and supernatants were incubated with anti-GFP antibody (Life Technologies) as indicated for 1 h at 4°C with gentle mixing of samples followed by the addition of 200 μl of fresh 50% (v/v) Protein A–Sepharose CL-4B beads and overnight incubation. The Protein A–Sepharose CL-4B beads were pelleted at 300 ***g*** for 3 min and washed four times with 1 ml of washing buffer containing 50 mM Tris/HCl (pH 7.5) and 150 mM NaCl. The protein was separated on a denaturing gel, immunoblotted on to a PVDF membrane, detected with anti-GFP antibody linked to horseradish peroxidase (Life Technologies) and visualized using the ECL detection system (GE Healthcare).

### In-gel digestion

To generate peptides for LC–ESI–MS/MS analysis, protein bands were excised from the SDS gels, washed with 500 μl of water for 15 min, and destained twice in 800 μl of 30% (v/v) acetonitrile for 30 min. Supernatants were discarded and gel slices were re-equilibrated with 500 μl of incubation buffer (100 mM ammonium bicarbonate, pH 8) for 10 min. After removal of the supernatants, the gel slices were dried by vacuum evaporation using a SpeedVac (Eppendorf) for 30 min at room temperature. For reduction of disulfide bonds, 116 μl of reducing solution (10 mM DTT) was added to the gel pieces and the samples were incubated at 56°C for 30 min, followed by cooling at 4°C for 10 min and a washing step with 150 μl of incubation buffer for 10 min. Alkylation of cysteine thiols was performed in the dark for 20 min by addition of 100 μl of 40 mM iodacetamide followed by quenching with 75 μl of reducing solution for 10 min and an additional washing step with 150 μl of incubation buffer for 15 min. To remove reducing and alkylation contamination, the samples were washed with 150 μl of incubation buffer for 5 min and 150 μl of pure acetonitrile was added to initiate gel shrinking. The samples were shaken for 15 min before the supernatant was removed, and dried using a SpeedVac for 30 min at room temperature before digestion.

The protein was digested with chymotrypsin, elastase, GluC and trypsin. Enzyme/protein ratios of 1:10 were used, except for trypsin which was used at 1:20. For the *in vitro* sample, all four enzymes were used in a multi-protease approach as described previously [30]. For the *in vivo* sample with a restricted amount of starting material, only the enzymes elastase and trypsin were used. Each protease stock solution was diluted in 50 mM ammonium bicarbonate, 5% (v/v) acetonitrile and 10 μl of the protease solution was added to the dehydrated gel slices. After incubating for 15 min, 90 μl of digestion buffer was added and the samples were left to digest overnight at 37°C. To extract the digested proteins, the supernatants were transferred to new tubes followed by two incubation steps with 150 μl of extraction buffer I [60% (v/v) acetonitrile and 0.5% TFA (trifluoroacetic acid)] and with 150 μl of pure acetonitrile for 15 min. The three extracts were combined, evaporated to dryness using a SpeedVac at room temperature, made up to 10 μl with loading buffer A [3% (v/v) acetonitrile and 0.1% TFA] and stored at −20°C before LC–ESI–MS/MS analysis.

### Phosphopeptide enrichment

Phosphopeptides from the *in vitro* sample were enriched using TiO_2_ beads (GL Sciences, MZ-Analysentechnik) similar to the method described previously [31]. First, 240 μg of TiO_2_ beads were washed twice with 20 μl of loading buffer [80% (v/v) acetonitrile, 15% (v/v) water, 5% (v/v) TFA and 1 M glycolic acid] for 15 min with shaking. The supernatant was discarded followed by peptide dilution in 50 μl of loading buffer. Diluted peptides were transferred and incubated with the TiO_2_ beads for 15 min while shaking. Beads were pelleted and the supernatant (NB fraction) was transferred to a new tube. Then, 50 μl of washing buffer I [80% (v/v) acetonitrile, 19% (v/v) water and 1% (v/v) TFA] was added and samples were shaken for 15 min. Beads were pelleted followed by a second washing step with washing buffer II [20% (v/v) acetonitrile, 79.8% (v/v) water and 0.2% TFA]. Combined supernatants of both washing steps were transferred to a new tube (W fraction). Before eluting the bound phosphopeptides, the beads were evaporated to dryness using a SpeedVac at room temperature for 30 min. Elution was performed twice using 50 μl and 100 μl of elution solution (1% NH_4_OH in H_2_O). Supernatants from both elution steps were combined (P fraction) and acidified with 5 μl of pure TFA and dried using a SpeedVac. Before MS analysis, each sample (NB, W or P) was diluted in 16.5 μl of eluent A (0.05% formic acid in water).

### Mass spectrometry

Protein digests were separated and analysed by RP (reverse-phase) LC using an UltiMate 3000 nano-HPLC system (Dionex) coupled online to an LTQ Orbitrap Velos equipped with ETD support (Thermo Fisher). Solvents used for LC were: solvent A (0.05% formic acid in water) and solvent B [80% (v/v) acetonitrile and 0.04% formic acid in water]. For sample loading, a solution of 3% (v/v) acetonitrile and 0.1% TFA was used. The flow rates were 30 μl/min for the loading and 30 nl/min for the micro-pump. Isocratic elution (5% B) was used for the first 4 min, followed by an increase to 10% B within 1 min. Then a linear gradient to 50% B within 80 min was used. Column washing was performed by a linear increase of B to 95% within 10 min and held for 5 min. Re-equilibration was set to 10 min at 5% B.

A dual top five CID (collision-induced dissociation)–HCD (higher-energy collisional dissociation fragmentation) method was written manually using XCalibur 2.1. Here, a MS full scan was acquired at a resolution of 60000 with activated pre-scan. For fragmentation, the five most intense precursor ions were selected for fragmentation by CID (default settings) and analysis in the ion trap. Afterwards, the same five most intense precursor ions were selected for fragmentation by HCD (normalized collision energy 45%; isolation window of *m*/*z* 3; remaining settings were default). To increase the number of acquired peptide ions, dynamic exclusion was activated (repeat count of three within 20 s and an exclusion duration of 30 s; the number of entries was set to 500). For LC–ESI–MS/MS analysis, 14 μl of the *in vivo* sample, 10 μl of the phosphopeptide fraction and 5 μl of the non-phosphopeptide fraction (*in vitro* sample) were injected.

### Database searches and data evaluation

Proteome Discoverer 1.4 (PD) with Mascot (version 2.2.04) and SEQUEST HT (shipped with PD) were used. The complete reference proteome set of *A. thaliana* was downloaded (November 2013, taxonomy identity: *A. thaliana*, reviewed proteins only) from Uniprot. A PD workflow consisting of the following nodes was created; if not specified otherwise, the default settings were used: (0) Spectrum Files; (1) Spectrum Selector; (2) MS2 - Spectrum Processor, (3) Scan Event Filter [splitting into CID-IT (CID in an ion trap) or HCD data]; (4) Mascot or SEQUEST HT (see below); (5) phosphoRS 3.0; (6) Percolator; (8) Scan Event Filter; (11) Event Detector and (12) Precursor Ions Area Detector. In total, four (independent) database searches were performed using CID-IT or HCD MS/MS data and Mascot and SEQUEST HT respectively. The resulting four .msf files were opened together and filtered for high confidence with a validation threshold of 0.01 based on the *q*-value. The combination of four independent searches was required as phosphoRS 3.0 [32] is only used for the search engine node with the lowest number in the PD workflow in a multi-database search engine workflow.

For PSMs (peptide-spectrum matches), the precursor mass tolerance window was set to 10 p.p.m. Fragment ions in MS/MS spectra were matched with a tolerance window of 0.02 Da and 0.3 Da for HCD (Orbitrap) and CID (ion trap). Carbamidomethylation was set as static, and oxidation of methionine and phosphorylation of serine, threonine and tyrosine as dynamic modification respectively. No-enzyme specificity was set as at least two (elastase and trypsin) and four (chymotrypsin, elastase, GluC and trypsin) different proteases were used respectively.

For data evaluation, GAMBAS (version 3) scripts were written in house. These scripts were used to group peptide identification features of the phosphopeptides identified. The .txt files of the PSMs were loaded and phosphorylated peptide identifications were grouped together on the basis of their sequence, retention time difference (<20 min) and [*M*+H]^+^ *m*/*z* values. Using these data, the start and end position of the peptide within the protein of interest was extracted. Furthermore, it was determined (Boolean value) whether this peptide was identified by Mascot or SEQUEST with CID and HCD respectively. Additionally, the number of hits for each of the four combinations was counted. The average peptide [*M*+H]^+^ mass, its average retention time and precursor mass (obtained from the Precursor Ions Area Detector) were calculated.

To allow for improved data handling of the high complex peptide composition with up to four enzymes that release peptides with nearly identical, but overlapping, sequences, the phosphorylation site mapping was performed by (i) using the results from the search engines, and (ii) the phosphoRS score. For the search-engine-based approach, (i), the number of phosphorylation site localizations was counted and the maximal value reported and set as ‘most likely’. In addition, the number of hits for this modification was set in relation to the overall number of peptide hits within the peptide under investigation. PhosphoRS report percentages were calculated by dividing the phosphoRS score by the accumulated value. Finally, the results were normalized to an accumulated value of 100%. The percentage values for each modification within a peptide under investigation were summed and divided by the number of hits. This value is reported and used as ‘normalized’ phosphoRS probability value.

## RESULTS AND DISCUSSION

### Identification of PSKR1 autophosphorylation sites

To understand phosphorylation-dependent signalling mechanisms of PSKR1, one aim of the present study was to identify phosphorylation sites in the cytoplasmic domain of the receptor using MS analysis. PSKRs belong to the large family of RD-type LRR RLKs, many of which are activated by phosphorylation of one or more residues in the AL (Figure 1). Regulation of receptor activity may also occur through phosphorylation of other subdomains. To identify phosphorylated amino acid residues in PSKR1, ectopically expressed His_6_-tagged soluble PSKR1 KD (His_6_–PSKR1-KD) protein was autophosphorylated *in vitro* as shown previously [16]. The protein was separated on an SDS gel and subjected to an in-gel multi-protease digestion procedure [30], using two relatively low-specific (chymotrypsin and elastase) and two high-specific (GluC and trypsin) proteases. Phosphopeptides were enriched using TiO_2_ and then analysed by LC–ESI–MS/MS. Two different MS/MS approaches, namely CID-IT and HCD in an Orbitrap mass analyser, were used, as the acquisition of a dual fragmentation method can increase the accuracy of phosphorylation site mapping utilizing computational proteomics tools [33].

On the basis of phosphopeptide grouping applied to estimate the correctness of the automated phosphorylation site mapping (see the Experimental section for further information), various phosphorylated peptides could be identified and their phosphorylation sites localized. Manual MS/MS spectra interpretation was done to verify the identifications.

Site mapping was significantly facilitated by using the four proteases and both the phosphoRS score [32] and the search engine results, e.g. for four peptides MSPYETHV, MSPYETHVSTDLVGTL, RLMSPYETHVSTDLVGTL and LMSPYETHVSTDLVGTLGYIPPEYGQASVATYK, covering the phosphorylation site Thr^890^ (Supplementary Figures S1A–S1D respectively). The use of four different proteases generated peptides ranging from 8 to 33 amino acids ([*M*+H]^+^: 1043.4–3667.7) with different physicochemical properties (e.g. change of fragmentation pattern by C-terminal residues). This enabled us to localize the phosphorylation site with high confidence. Overall, using this multi-protease strategy, a sequence coverage of 99% was achieved for the His_6_–PSKR1-KD protein.

**Figure 2:**
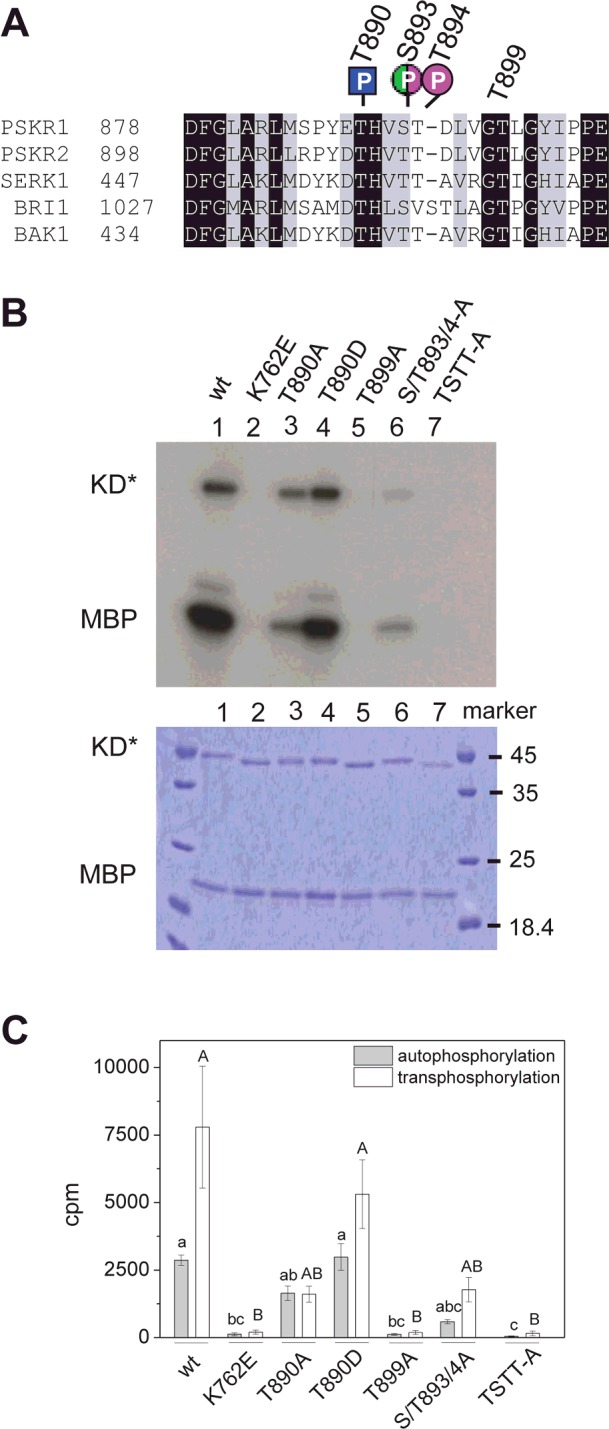
Site-directed mutagenesis of PSKR1 activation segment or KD residues impairs kinase activity *in vitro* (**A**) Alignment of the activation segments encompassing the AL (Ser^886^–Thr^894^ in PSKR1) of PSKR1, PSKR2, SERK1, BRI1 and BAK1. The phosphorylation sites of PSKR1 that were identified *in vitro* (magenta), *in planta* (green) or in the kinase-inactive variant in *E. coli* (blue) are indicated. (**B**) Top: autoradiograph of recombinant PSKR1-KD variants (KD*) showing autophosphorylation of KD* and *trans*-phosphorylation of MBP: wild-type (lane 1), the kinase-inactive His_6_–PSKR1(K762E)-KD (lane 2), His_6_–PSKR1(T890A) (lane 3), His_6_–PSKR1(T890D) (lane 4), His_6_–PSKR1(T899A) (lane 5), His_6_–PSKR1(S893A/T894A) (lane 6), and His_6_–PSKR1(TSTT-A) (lane 7). Bottom: Coomassie Blue-stained gel as a control for protein loading. (**C**) Mean±S.E.M. radioactivity (*n*=6 from three independent experiments) of *trans*-phosphorylated [^32^P]MBP and autophosphorylated [^32^P]KD* is given as c.p.m. Kruskal–Wallis all-pairwise comparisons were performed for autophosporylation (lower-case letters) and for *trans*-phosphorylation (capital letters) activities. TSTT-A, T890A/S893A/T894A/T899A; wt, wild-type.

On the basis of the dataset and on the number of PSM and phosphoRS scores, eight phosphorylation sites were identified: Ser^717^ in the JM domain, Ser^733^, Thr^752^, Ser^783^, Ser^864^, Ser^911^, Ser^958^ and Thr^998^ (Figure 1 and Table 1). Moreover, on the basis of automated and manual inspection of the data, at least one and possibly two sites (Thr^890^, Ser^893^ or Thr^894^) in the AL of PSKR1 spanning residues 886–894 is likely to be phosphorylated (Figures 1 and 2A, and Table 1). Here, Thr^890^ represented the most likely phosphorylation site on the basis of the automated phosphorylation site mapping approaches (phosphoRS score and database search engines). In addition, peptides covering this potential phosphorylation site show a high diversity of sequence length (between 8 and 33 amino acids) and starting positions (883–886). This resulted in 29 unique peptide sequences encompassing different modification forms of a peptide with the same sequence that, as a group, can be used for phosphorylation site localization. Together with these singly phosphorylated peptide species, a second, doubly phosphorylated, form was also detected, probably a combination of Thr^890^ and Ser^893^ or Thr^890^ and Thr^894^. However, owing to the close proximity of Ser^893^ and Thr^894^, an unambiguous localization of the second site was not possible. At present, we cannot exclude that both forms exist in parallel.

**Table 1:** Identification of *in vitro* phosphorylation sites of His_6_–PSKR1-KD protein by LC–ESI–MS/MS

Residue	Median phosphoRS score	Average phosphoRS score	Standard error	Number of unique* peptides	Number of accumulative PSMs†
Ser^717^	100.00	88.69	7.47	9	134
Ser^733^	48.30	48.30	–	1	28
Thr^752^	100.00	100.00	–	1	2
Ser^783^	58.40	58.40	–	1	11
Ser^864^	98.20	98.20	–	1	5
Thr^890^	95.95	86.99	2.44	48	1306
Thr^890^ and Ser^893^/Thr^894^	Ambiguous	Ambiguous	Ambiguous	9	142
Ser^911^	100.00	100.00	–	1	9
Ser^958^	89.80	79.95	5.97	11	99
Thr^998^	61.15	61.15	11.15	2	3

*Unique peptides are identified peptide sequences which can be used for unambiguous peptide and protein identification. For the multi-protease approach applied in the present study, this also includes overlapping peptide sequences.

†PSMs are MS/MS spectra to which a peptide sequence was mapped by a database search algorithm and validated further by false discovery rate analysis (1%). Here, the total number of PSMs was summed for each individual phosphorylation site.

To verify that the identified amino acids were indeed targets of autophosphorylation by PSKR1 rather than targets of *E. coli* kinases we expressed and affinity-purified kinase-inactive His_6_–PSKR1(K762E)-KD protein [16]. In this mutant protein, an invariant lysine residue in subdomain II that is conserved in all kinases and that is essential for kinase activity was replaced by glutamate [34]. Kinase-inactive His_6–_PSKR1(K762E)-KD protein was subjected to a phosphorylation assay and phosphopeptide analysis by LC–ESI–MS/MS. The sequence coverage of His_6_–PSKR1(K762E)-KD was comparable with that obtained for His_6_–PSKR1-KD. For this mutant protein, one phosphorylation site (Thr^890^) was determined *in vitro* (median phosphoRS score >99%; Supplementary Table S1) suggestive of *trans*-phosphorylation of this residue by *E. coli*.

To compare the precursor ion signal areas and the PSM respectively, as a quantitative measure of the extent of phosphorylation, the corresponding values (i.e. precursor area or PSM) of the sample were divided by the sum of sample and control. In total, 11 peptide species (including forms with different modifications) were identified for the phosphorylation site Thr^890^ (Supplementary Table S2). To normalize the data, the median of all 11 peptide species was calculated. For the precursor area, median values of 94.5% (His_6_–PSKR1-KD) and 5.5% [His_6_–PSKR1(K762E)-KD] was obtained. Similar values were calculated for the PSM [89.7% for His_6_–PSKR1-KD and 10.3% for His_6_–PSKR1(K762E)-KD]. To evaluate the extent of phosphorylation between His_6_–PSKR1-KD and His_6_–PSKR1(K762E)-KD, the same approach as described before was performed. Comparing the peptide sequences, the percentage distribution [His_6_–PSKR1-KD/(His_6_–PSKR1-KD+His_6_–PSKR1(K762E)-KD] was 94% and 90% for the label-free and SPC (spectral counting) approach respectively (Supplementary Table S2), indicating that the extent of phosphorylation in His_6_–PSKR1(K762E)-KD was much lower. Nonetheless, the kinase-inactive His_6_–PSKR1(K762E)-KD protein is a suitable substrate for an as yet unknown bacterial kinase. Probably, phosphorylation takes place during ectopic expression of the mutant protein in *E. coli*. It is unlikely that the phosphorylation event originates from any kinase activity of the mutant His_6_–PSKR1(K762E)-KD protein, since a replacement of the invariant lysine residue in subdomain II by glutamate results in a loss of kinase-mediated phosphotransfer [34]. Consistent with this, His_6_–PSKR1(K762E)-KD kinase activity was essentially abolished (Figure 2). Phosphorylations at the JM domain, the KD and close to the CT domain result from activity of the wild-type His_6_–PSKR1-KD protein and reveal a pattern that is common for receptor kinases [21,23].

### Identification of *in planta* PSKR1 phosphorylation sites

**Figure 3:**
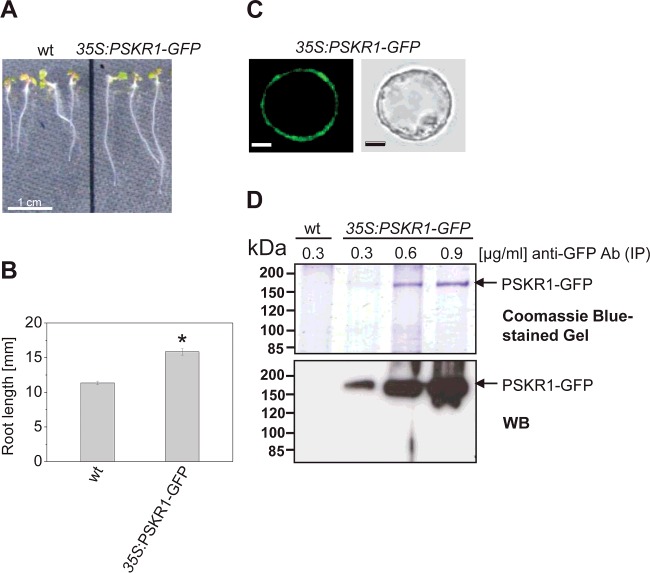
Functional analysis, localization and purification of PSKR1–GFP (**A**) Phenotypes of 5-day-old seedlings from wild-type and *35S:PSKR1-GFP* expressed in the wild-type background. (**B**) Mean±S.E.M. root lengths were determined in two independent experiments with at least 46 seedlings analysed per genotype. The asterisk indicates significantly different values between wild-type and *35S:PSKR1-GFP* (*P*<0.05, two-sample Student's *t* test). (**C**) PSKR1–GFP localization was analysed by confocal laser-scanning microscopy in mesophyll protoplasts of 4-week-old soil-grown *35S:PSKR1*-*GFP* plants. The receptor protein is expressed and localized at the plasma membrane. Scale bars, 5 μm. (**D**) Immunoprecipitation of PSKR1–GFP from total protein extracts obtained from seedlings expressing PSKR1–GFP with anti-GFP antibody added at the concentrations indicated. As a control, extract from wild-type seedlings was analysed. The proteins immunoprecipitated from wild-type and PSKR1–GFP extracts were separated by SDS/PAGE (10% gel) and fusion protein was detected with an anti-GFP antibody. Molecular masses of marker proteins are indicated in kDa. Ab, antibody; IP, immunoprecipitation; WB, Western blot; wt, wild-type.

To identify *in planta* PSKR1 phosphorylation sites, a C-terminally GFP-tagged full-length PSKR1 sequence was generated, placed under control of the CaMV 35S promoter and stably expressed in wild-type plants. PSKR1–GFP fluorescence was observed in mesophyll protoplasts derived from 4-week-old *35S:PSKR1-GFP* plants where it was localized to the plasma membrane as expected (Figure 3C). Roots of 5-day-old *35S:PSKR1-GFP* seedlings were significantly longer than wild-type roots indicating that receptor overexpression caused a specific growth response (Figures 3A and 3B) and that the GFP fusion did not impair *in planta* receptor function. Total protein was extracted from 14-day-old *35S:PSKR1-GFP* and from wild-type seedlings followed by immunoprecipitation with anti-GFP antibody. A band with a size of approximately 175 kDa was detected in protein samples of the transgenic line, but not in the sample from wild-type seedlings (Figure 3D). GFP has a molecular mass of ∼27 kDa and the *Arabidopsis* PSKR1 protein has a calculated mass of 112353 Da. However, the PSKR from *Daucus carota* was shown to be glycosylated *in planta*, increasing its molecular mass by ∼10 kDa. The ∼175 kDa protein band was detected in Western blot analysis with anti-GFP antibody, suggesting that it corresponded to the PSKR1–GFP fusion protein. This was subsequently verified by LC–ESI–MS/MS. The amount of immunoprecipitated PSKR1–GFP protein correlated with the anti-GFP antibody concentration that was used for immunoprecipitation.

To identify *in planta* PSKR1 phosphorylation sites, the PSKR1–GFP band was excised, in-gel digested with trypsin or elastase and subjected to LC–ESI–MS/MS analysis. The phosphorylated peptides identified are listed in Supplementary Table S3 and representative MS/MS spectra are shown in Supplementary Figures S2A–S2C. Combining the peptides generated by elastase and tryptic digestion, 74.70% of the protein sequence was covered. Three possible phosphorylation sites were identified. Ser^698^ (median phosphoRS score >93%; Figure 1A and Supplementary Table S3), which is located in the JM region represents a true *in planta* phosphorylation site. A double-phosphorylated peptide probably at Ser^696^ and Ser^698^ was also detected. A third site was located at Ser^886^ or Ser^893^ (Figures 1A and 2A, and Supplementary Table S3). Unfortunately, an unambiguous localization of this phosphorylated residue was not possible. More residues may be phosphorylated *in planta* that may be identified using the native promoter or an optimized extraction procedure. Nonetheless, the data indicate that at least one site (Ser^886^ or Ser^893^) in the AL of PSKR1 is phosphorylated *in planta*.

Taken together, the LC–ESI–MS/MS analyses identified only phosphorylation of serine/threonine residues consistent with the fact that PSKR1 displays a predicted serine/threonine kinase catalytic domain based on the 12 subdomains that are characteristic of this class of kinases [9,14,15]. A preference for serine over threonine residues was observed which has also been shown for the LRR RLKs BRI1 and CLV1 [35–37]. A recent study found that BRI1, BAK1 (BRI1-ASSOCIATED RECEPTOR KINASE) and BKK1 (BAK1-LIKE 1) are also autophosphorylated at tyrosine residues, indicating that these are dual-specificity kinases [38]. Bojar et al. [39] report conservation of the conformation of the AL and of core phosphorylation sites among different plant RLKs [40–44]. It is hence conceivable that PSKR1 could also exhibit dual specificity, although phosphorylation of tyrosine residues was not detected in the present study.

### Conservation of serine/threonine residues in positions aligning with phosphosites in PSKR1

**Figure 4:**
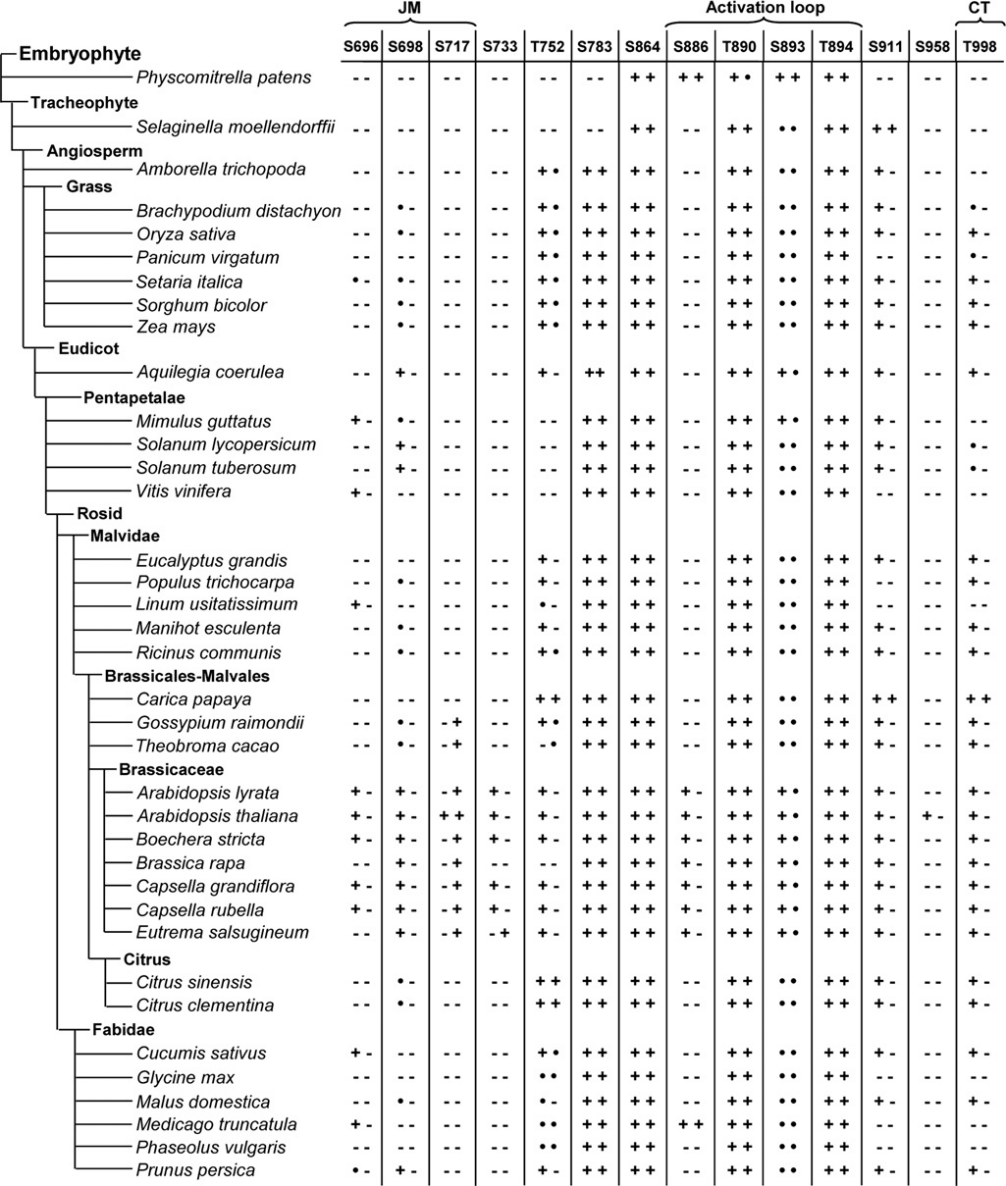
Conservation of serine and threonine residues of PSKR1 orthologues (left symbol) and PSKR2 orthologues (right symbol) in positions aligning with PSKR1 phosphorylation sites A dot indicates a conserved substitution of the respective residue by serine, threonine or tyrosine;–indicates no conservation, and + indicates a conserved P site. The species tree used in the present study was constructed by modifying the Phytozome version 10 plant tree of life.

To identify conserved positions of phosphorylatable amino acids in PSKRs throughout the plant kingdom, we identified PSKR1 as well as PSKR2 orthologues from the genomes of 35 higher plants and of two mosses using the Phytozome database [45]. The intracellular kinase sequences were aligned (Supplementary Figures S4 and S5). The conservation of phosphorylatable amino acids at each site of PSKR1 and PSKR2 orthologues is summarized in Figure 4. Three of the positions that are phosphorylated in the AL of PSKR1, Thr^890^, Ser^893^ and Thr^894^, were conserved in all PSKRs including those from mosses. The ambiguous phosphorylation site at Ser^886^ was conserved in PSKR1 homologues from Brassicaceae only. A serine residue aligns with Ser^864^ in subdomain VIb N-terminal to the activation segment in all plant PSKR1 and PSKR2 homologues, indicative of a conserved function in PSKRs.

In the JM region, the position of at least one of the two residues Ser^696^ and Ser^698^ was conserved in 83.3% of higher plant PSKR1 orthologues, but not in PSKR2 orthologues. In PSKR1, Ser^717^ was exclusive to *A. thaliana*, whereas Ser^717^ was present in ∼22% of higher plant PSKR2 orthologues, mostly from the Brassicaceae lineage.

Subdomain X is overall little conserved and Ser^958^ that was phosphorylated in PSKR1 was present at this position only in that receptor. By contrast, Thr^998^ was conserved in 83.3% of PSKR1 orthologues, but in only one PSKR2 protein. The positions of the phosphorylated serine residues in the JM region of PSKR1 are not conserved in the two moss homologues, whereas, in most higher plant orthologues, at least one of the two positions is occupied by a phosphorylatable amino acid. Taken together, the positions of phosphorylatable amino acids in the AL are strictly conserved in PSKRs, suggesting a conserved mode of receptor regulation. By contrast, positions of PSKR1 phosphosites in the JM region are not retained in PSKR2 orthologues and are altogether absent from PSKR orthologues from mosses. Possibly, regulation via phosphorylation of the JM region has evolved early during higher plant evolution, possibly arising from a need for more complex receptor regulation in cormophytes. The differences in phosphosites between PSKR1 and PSKR2 orthologues suggest that regulation at these sites has evolved faster and is less subject to selection pressure than the regulation of the AL.

To date, regulation of only a few of the 610 RLKs encoded in the *Arabidopsis* genome [46] has been analysed and therefore our knowledge on conserved and unique modes of plant receptor regulation at the level of phosphorylation is limited [24,37,47–49]. The detailed analysis provided in the present study not only helps to further our understanding of PSK signalling, but also provides comparative data for the study of related RLKs.

### Phosphoablative mutagenesis suggests that phosphorylation within the AL activates PSKR1

The activation segment starts at the highly conserved DFG motif in kinase subdomain VII and most often terminates with the sequence APE in subdomain VIII [14,15,22] (Figures 1 and 2A). It consists of the Mg^2+^-binding loop, the β-9 sequence, the AL (Ser^886^–Thr^894^ in PSKR1) and the p+1 loop with Thr^899^ in PSKR1 [50]. Phosphorylation of one to three residues in the AL results in a conformational change accompanied by kinase activation. In some cases, it also affects binding of kinase substrates [22,23]. To resolve the ambiguities concerning specific *in vitro* phosphorylation sites in the AL of PSKR1 and to test for a functional role of these phosphorylation sites with respect to kinase activity, site-directed mutagenesis was performed on the soluble KD of PSKR1 (PSKR1-KD). The serine and threonine residues were mutated to alanine which prevents phosphorylation and, in the case of Thr^890^, to aspartate which acts as a phosphomimic [23,36]. PSKR1-KD proteins with T890A, T890D, T899A and S893A/T894A mutations, or the quadruple mutation T890A/S893A/T894A/T899A (denoted TSTT-A), were ectopically expressed as fusion proteins with an N-terminal His_6_ tag. Auto- and *trans*-phosphorylation of the common substrate MBP were analysed *in vitro* using radiolabelled [γ-^32^P]ATP. As controls, the wild-type His_6_–PSKR1-KD and the His_6_–PSKR1(K762E)-KD variant were analysed [16]. Even though Thr^890^ was a target of *trans*-phosphorylation by *E. coli*, it cannot be ruled out that it is also a target of autophosphorylation. On the basis of this reasoning, we included analysis of this residue. The T890A mutation resulted in overall reduced kinase activity compared with the wild-type, whereas the T890D mutant displayed wild-type activity (Figures 2B and 2C). The auto-, but not *trans*-, phosphorylation activity of the S893A/T894A mutant was lower than that of the T890A mutant.

Several studies described an important role for AL phosphorylation in the kinase activity of BRI1 and BAK1 [24,25,41,44]. Mutations of BRI1 AL residues corresponding to PSKR1 Thr^890^ and Ser^893^ likewise had a moderate effect on BRI1 kinase activity, indicating that the kinase activities of soluble PSKR1-KD and BRI1-KD are regulated in a similar manner by this residue. BAK1-KD is also autophosphorylated at two residues equivalent to Thr^890^ and Ser^893^. However, single mutations of BAK1 AL residues corresponding to PSKR1 Thr^890^, Ser^893^ and Thr^894^ had little effect on BAK1 kinase activity, revealing a distinct difference between this co-receptor and the receptors BRI1 and PSKR1. Replacement of all three threonine residues by alanine in the AL of BAK1 abolished its activity.

Interestingly, BRI1 and SERK1 AL residues corresponding to PSKR1 Thr^890^ and Ser^893^ affect substrate phosphorylation more than autophosphorylation *in vitro* [24,25]. On the basis of the crystal structure of the BRI1 KD, the BRI1 AL residues corresponding to PSKR1 Thr^890^ and Ser^893^ were found to be phosphorylated and were surface-oriented, probably contributing to the stabilization of the conformation of the AL by interaction with a third residue equivalent to PSKR1 His^891^ [41]. In the dephosphorylated state, the AL impairs ATP binding and/or lowers the rate of phosphotransfer to the substrate [21,22]. Dependent on its phosphorylation state, the AL is characterized by defined conformational changes [23,37]. Our data suggest that differential phosphorylation of the AL may result in partial receptor activation.

Thr^899^ was not identified as a site of phosphorylation. Nonetheless, it is conserved in all PSKR1 and PSKR2 orthologues from higher and lower plants (Figure 4, and Supplementary Figures S4 and S5) and the equivalent residue in BRI1 was shown to be a likely candidate for phosphorylation [24]. Thr^899^ was therefore included in this mutational analysis. The T899A mutant was inactive, strongly arguing for a crucial role of the invariant Thr^899^ in kinase activity. Similarly, the quadruple TSTT-A mutation resulted in loss of kinase activity.

### Phosphoablative mutagenesis within the AL differentially alters PSKR1 activity *in planta*

The high conservation of potential phosphosites in the AL of PSKR orthologues and the differential effect of individual phosphosites on PSKR1 kinase activity prompted us to analyse the impact of AL phosphorylation and of the adjacent Thr^899^ on growth regulation by PSKR1 *in planta*. To that end, we generated T890A, T890D, T899A, S893A/T894A and TSTT-A point-mutated full-length *PSKR1* sequences that were expressed under the control of the CaMV 35S promoter in the receptor-null background. Expression of the transgenes was analysed in 5-day-old seedlings in the independent lines *35S:PSKR1*(*T890A*)-*1*,*2*,*3*,*4*; *35S:PSKR1*(*T899A*)-*1*,*2*,*3*,*4*,*5*, *35S:PSKR1*(*S893A*/*T894A*)-*1*,*2*,*3*,*4*,*5*, *35S:PSKR1*(*TSTT*-*A*)-*1*,*2*,*3* and *35S:PSKR1*(*T890D*)-*1*,*2*,*3*,*4*,*5* by RT–PCR (Supplementary Figures S3A and S3B). In the null background line *pskr1-3 pskr2-1* (denoted *r1r2*), no *PSKR1* mRNA was detected, indicating that growth promotion was driven exclusively by the respective receptor variant.

**Figure 5:**
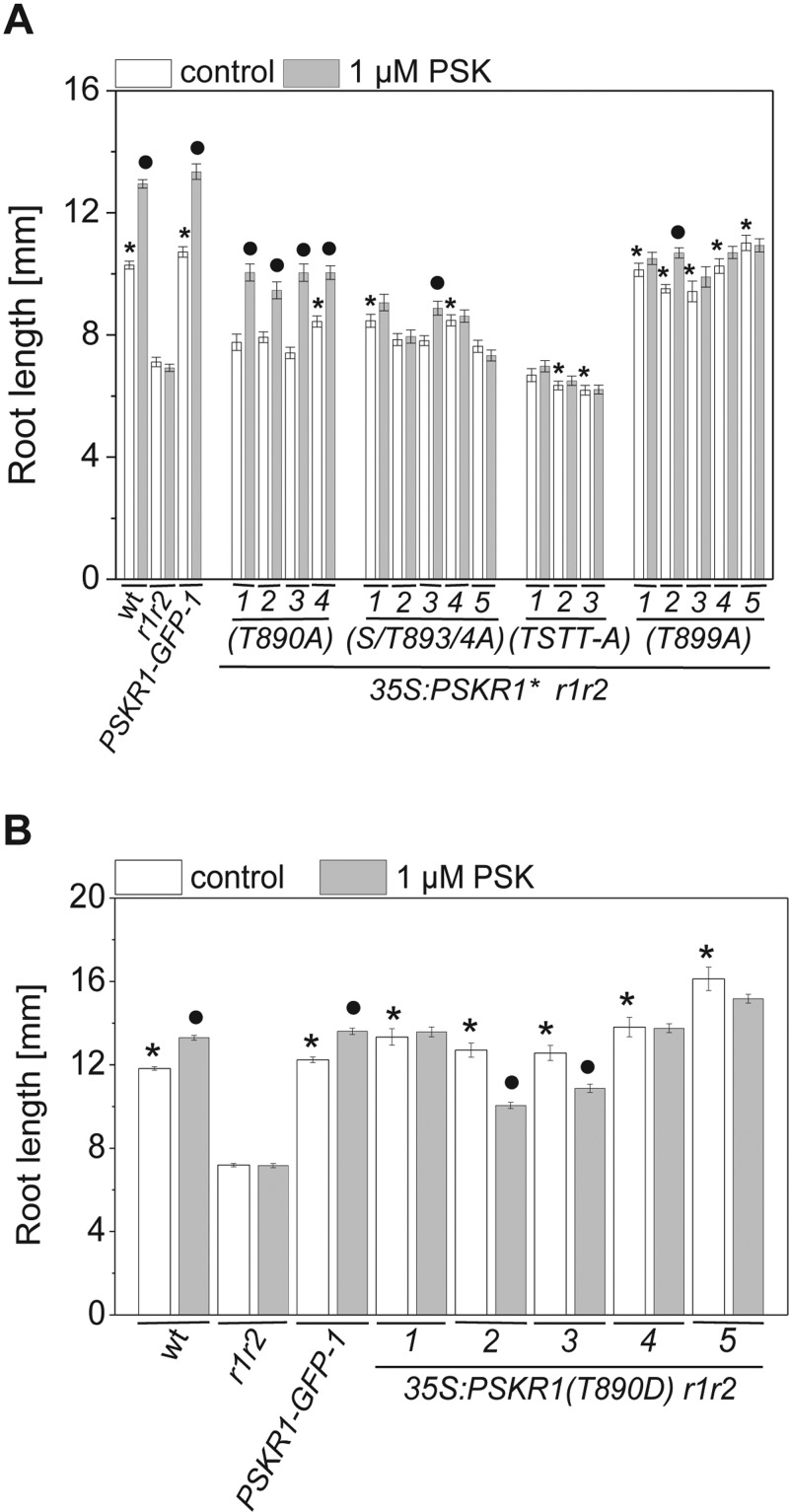
Inhibition of phosphorylation of specific activation segment residues through site-directed mutagenesis differentially impairs growth signalling by PSKR1 in seedling roots (**A**) Seedlings of up to five independent transgenic lines of the PSKR1(T890A), PSKR1(S893A/T894A), PSKR1(T899A), and PSKR1(TSTT-A) mutants in the receptor-null *r1r2* background were grown with or without 1 μM PSK for 5 days. Mean±S.E.M. root lengths were determined in three independent experiments with at least 48 seedlings analysed per genotype. Asterisks indicate significantly different values between *r1r2* and the lines expressing different receptor variants (*P*<0.001, two-sample Student's *t* test). Circles indicate significantly different values between treatments for each genotype (*n*≥32; *P*<0.001). (**B**) Mean±S.E.M. root lengths were determined in five independent lines of the phosphomimic receptor variant PSKR1(T890D) expressed in the receptor-null *r1r2* background. At least 57 seedlings were analysed per genotype in three independent experiments in the absence or presence of 1 μM PSK. Asterisks indicate significantly different values between *r1r2* and the PSKR1(T890D) lines (*P*<0.001, two-sample Student's *t* test). Circles indicate significantly different values between treatments for each PSKR1(T890D) line (*n*≥50; *P*<0.001). S/T893/4A, S894A/T894A; wt, wild-type.

Transgenic lines expressing the various receptor mutants were analysed for their ability to rescue the short-root phenotype of the PSKR-null mutant *r1r2*. Root lengths of 5-day-old wild-type, *r1r2*, *r1r2 35S:PSKR1*-*GFP*-*1*, *r1r2 35S:PSKR1*(*T890A*), *r1r2 35S:PSKR1*(*T890D*), *r1r2 35S:PSKR1*(*T899A*), *r1r2 35S:PSKR1*(*S893A*/*T894A*) and *r1r2 35S:PSKR1*(*TSTT*-*A*) seedlings as well as their responsiveness to exogenously supplied PSK were measured (Figures 5A and 5B). *r1r2* roots were significantly shorter than wild-type roots. Expression of wild-type PSKR1 in *r1r2 35S:PSKR1*-*GFP*-*1* seedlings rescued the short-root phenotype of *r1r2* and resulted in root lengths similar to that of wild-type.

The phosphoablative mutations in the AL and of the adjacent Thr^899^ impaired root elongation in different ways. The T890A mutant did not, or only weakly, rescued the short-root phenotype of *r1r2* in accord with the reduced kinase activity of this mutant (Figures 2B and 5A). However, *r1r2 35S:PSKR1*(*T890A*)-*1*,*2*,*3*,*4* seedlings were still responsive to PSK, indicating that activation of the receptor by ligand binding is not fully dependent on Thr^890^. Replacing Thr^890^ by a phosphomimic aspartate residue resulted in wild-type root lengths in five independent lines (Figure 5B). At least two of the *r1r2 35S:PSKR1*(*T890D*) lines (4 and 5) had an overexpression phenotype [4], indicative of an active receptor. None of the lines displayed enhanced root elongation when treated with PSK, possibly because the response was saturated. The PSKR1(S893A/T894A) receptor had weak root-growth-promoting activity in accord with the weak *in vitro* kinase activity (Figures 2B and 5A). Root lengths were intermediate between those of the receptor-null mutant and the wild-type (Figure 5A). Unlike the PSKR1(T890A) receptor, which was activated by PSK, ablation of the phosphosites Ser^893^/Thr^894^ abolished responsiveness to PSK in four of the five PSKR1(S893A/T894A) lines and diminished it considerably in the fifth line.

An intriguing difference between *in vitro* kinase activity and *in planta* receptor function was unveiled for the PSKR1(T899A) mutant. Even though this residue was not identified as being phosphorylated, it is a conserved residue in all PSKRs, and the BRI1 AL residue corresponding to PSKR1 Thr^899^ is a likely phosphosite *in planta* [24]. Mutation of the conserved AL residue in BRI1 and BAK1 that corresponds to Thr^899^ in PSKR1 abolished the kinase activities of both receptors. Whereas the T899A mutation in PSKR1-KD similarly abolished kinase activity *in vitro* (Figures 2B and 2C), it did not impair basal PSKR1 receptor activity *in planta* (Figure 5A). This suggests that folding of the KD in an active receptor *in planta* may depend on other factors. It is conceivable that the PSKR1(T899A) receptor is complemented by heterodimerization with another kinase *in planta* that aids in signal transduction as demonstrated recently for BAK1 [20]. The PSKR1(T899A) seedlings were, however, unresponsive to PSK, indicating some impairment in signal regulation.

Mutating all three serine and threonine residues in the AL and Thr^899^ to alanine not only abolished kinase activity, but also, upon *in planta* expression, resulted in roots that were as short as or shorter than the roots of *r1r2* seedlings, indicating that this receptor was inactive *in planta* (Figure 5A). The importance of these AL residues to receptor activity in planta is supported by the fact that all sites are under high selection pressure in PSKR1 and PSKR2 orthologues.

**Figure 6:**
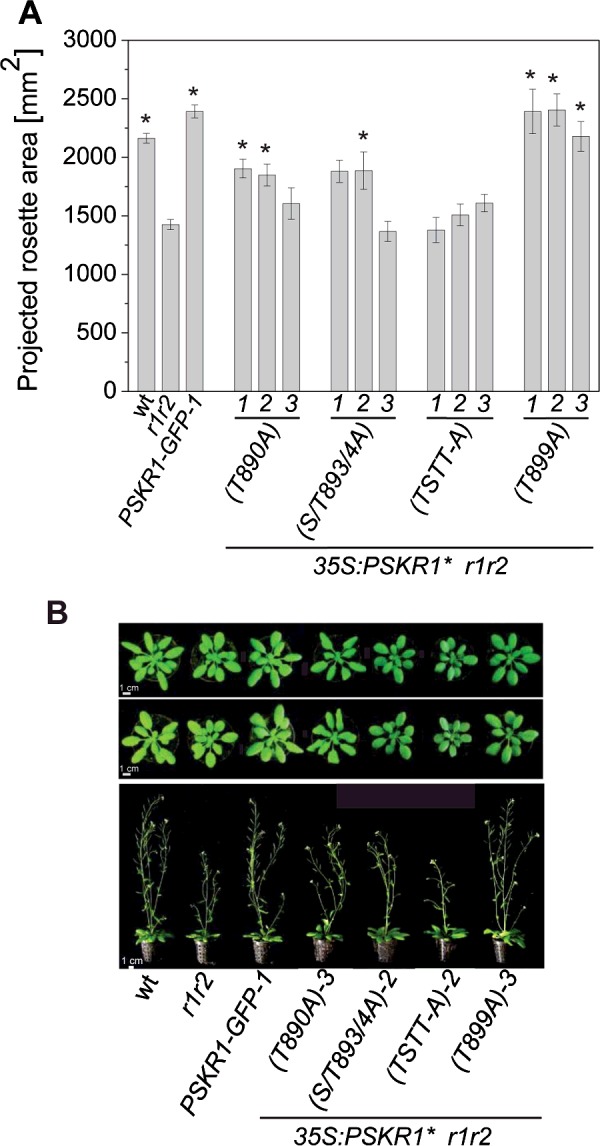
Preventing phosphorylation of specific activation segment residues impairs shoot growth (**A**) The mean±S.E.M. projected rosette area from PSKR1(T890A), PSKR1(S893A/T894A), PSKR1(T899A) and PSKR1(TSTT-A) plants (*n*=8–9) was calculated using the program Rosette Tracker [28]. Wild-type plants and plants complemented with a GFP-tagged wild-type receptor in the receptor-null background were included as controls. Asterisks indicate significantly different values between *r1r2* and the receptor variants (*P*<0.05, two-sample Student's *t* test). (**B**) Representative phenotypes of 4-week-old (upper row) and of 6-week-old (lower row) soil-grown plants from wild-type, *r1r2*, *35S:PSKR1*-*GFP*-*1*, *35S:PSKR1*(*T890A*)-*3*, *35S:PSKR1*(*S893A*/*T894A*)-*2, 35S:PSKR1*(*TSTT*-*A*)-*2* and *35S:PSKR1*(*T899A*)-*3*. Scale bars, 1 cm. S/T893/4A, S894A/T894A; wt, wild-type.

Analysis of soil-grown plants revealed that the phosphoablative mutations had a similar effect on plant shoot growth as it had on seedling root growth and hence confirmed the differential effect of specific phosphosites on receptor activity (Figure 6). The rosette sizes of 4-week-old *r1r2 35S:PSKR1*(*T890A*) and of *r1r2 35S:PSKR1*(*S893A*/*T894A*) plants as well as the flower stalks of 6-week-old plants were intermediate between *r1r2* and wild-type plants (Figure 6A and 6B). The rosette sizes of *r1r2 35S:PSKR1*(*T899A*) plants had wild-type size as was observed for seedling roots (Figures 5A and 6A). And as with seedling roots, the rosettes and flower stalks of *r1r2 35S:PSKR1*(*TSTT*-*A*)-*1*,*2*,*3* plants were no bigger than those of *r1r2*. Hence the concerted phosphoablative mutations in the activation segment of PSKR1 resulted in the inability of the PSKR1(TSTT-A) receptor to rescue the short-root and small-shoot phenotype of receptor-null plants.

In summary, our results showed that PSKR1 belongs to the RD-type kinases that require phosphorylation within the AL for their full activation. Phosphoablative mutations of one or two AL phosphosites partially reduced kinase activity and receptor function, but did not abolish it. We hypothesize that differential phosphorylation of AL residues of PSKR1 can modulate receptor activity.

## Abbreviations

AL: activation loop
BAK1: BRI1-ASSOCIATED RECEPTOR KINASE
BRI1: BRASSINOSTEROID-INSENSITIVE 1
CaMV: cauliflower mosaic virus
CID: collision-induced dissociation
CID-IT: CID in an ion trap
CV1: CLAVATA1
CT: C-terminal
HCD: higher-energy collisional dissociation fragmentation
JM: juxtamembrane
KD: kinase domain
LRR RLK: leucine-rich repeat receptor-like kinase
MBP: myelin basic protein
PSK: phytosulfokine
PSKR: phytosulfokine receptor
PSM: peptide-spectrum match
RT: reverse transcription
TFA: trifluoroacetic acid
